# A Novel Noise Reduction Approach of Acoustic Emission (AE) Signals in the SiC Lapping Process on Fixed Abrasive Pads

**DOI:** 10.3390/mi15070900

**Published:** 2024-07-10

**Authors:** Jie Lin, Jiapeng Chen, Wenkun Lin, Anjie He, Xiaodong Hao, Zhenlin Jiang, Wenjun Wang, Baoxiu Wang, Kerong Wang, Ying Wei, Tao Sun

**Affiliations:** 1 Research Center for Advanced Micro-/Nano-Fabrication Materials, School of Chemistry and Chemical Engineering, Shanghai University of Engineering Science, Shanghai 201620, China; m340121117@sues.edu.cn (J.L.); tele19804050865@163.com (W.L.); 18616158730@163.com (A.H.); wzhwblhm@163.com (X.H.); jiangzhenlin@sues.edu.cn (Z.J.); 04200004@sues.edu.cn (W.W.); bxwang_618@163.com (B.W.); 2 State Key Laboratory of Silicon and Advanced Semiconductor Materials, Zhejiang University, Hangzhou 310027, China; 3 Jiangsu Key Laboratory of Precision and Micro-Manufacturing Technology, National Key Laboratory of Science and Technology on Helicopter Transmission, College of Mechanical and Electrical Engineering, Nanjing University of Aeronautics and Astronautics, Nanjing 210016, China; jhcwkr@163.com; 4 Zhengzhou Abrasive Grinding Research Institute Co., Ltd., State Key Laboratory for High Performance Tools, Zhengzhou 450001, China; 18317575915@163.com; 5 Mechanical & Electrical Engineering College, Jinhua Polytechnic, Jinhua 321000, China

**Keywords:** acoustic emission, noise reduction, frequency threshold, wavelet packet, spectral subtraction, fixed abrasive lapping

## Abstract

Acoustic emission (AE) technology has been widely utilized to monitor the SiC wafer lapping process. The root-mean-square (RMS) of the time–domain eigenvalues of the AE signal has a linear relationship with the material removal rate (MRR). However, the existence of background noise severely reduces signal monitoring accuracy. Noise interference often leads to increased RMS deviation and signal distortion. In the study presented in this manuscript, a frequency threshold noise reduction approach was developed by combining and improving wavelet packet noise reduction and spectral subtraction noise reduction techniques. Three groups of SiC lapping experiments were conducted on a fixed abrasive pad, and the lapping acoustic signals were processed using three different noise reduction approaches: frequency threshold, wavelet packet, and spectral subtraction. The results show that the noise reduction method using the frequency threshold is the most effective, with the best coefficient of determination (R^2^) for the linear fit of the RMS to the MRR.

## 1. Introduction

SiC wafers are third-generation semiconductors that offer a high critical breakdown electric field and electron mobility, making them ideal for use in railroad traction inverters and electric vehicle power devices [1,2,3,4]. SiC substrates are frequently required to be flattened by free abrasive and fixed abrasive lapping before they can be used to make power devices. However, due to its brittleness and high hardness, SiC processing is very challenging. Compared to free abrasive lapping, fixed abrasive lapping offers higher removal efficiency, and less environmental pollution, and is widely used in difficult-to-machine material processing [5,6,7,8,9,10]. In addition, process monitoring is an essential aspect of the processing procedure. The primary method is online monitoring [11].

Friction-based [12], polishing pad temperature-based [13], optical-based [14,15], and acoustic emission-based monitoring methods [16] are the primary online monitoring methods for lapping processes. Compared to other monitoring methods, AE technology presents high sensitivity and applicability and other advantages because the acoustic signals are easy to collect and can reflect the processing status in real time to provide effective information for processing guidance. Ahn et al. [17] utilized the AE technique and atomic force microscopy (AFM) to differentiate the material deformation state during silicon wafer nano-scribing and found that the RMS can effectively reflect the deformation state. Wei et al. [18] utilized the AE technique to monitor the polishing process of sapphire wafers to establish a linear regression relationship between RMS and MRR and to predict the MRR from the RMS of AE signals.

High-sensitivity AE technology ensures a comprehensive collection of processing signals but also results in the collection of more noise. Therefore, noise reduction is necessary to improve the accuracy of the processed acoustic signals. Commonly used methods for reducing noise in acoustic signals include wavelet noise reduction [19,20], wavelet packet noise reduction [21,22], and spectral subtraction noise reduction [23,24]. Wavelet noise reduction and wavelet packet noise reduction involve processing the wavelet coefficients, while spectral subtraction noise reduction is a process that occurs in the frequency domain. Sikder et al. [25] monitored the friction coefficient and AE signals during chemical–mechanical polishing and used wavelet noise reduction to reduce invalid signals to obtain more accurate signal characterization.

This manuscript develops a frequency threshold noise reduction method by combining and improving wavelet packet noise reduction and spectral subtraction noise reduction. Experimental evidence indicates that frequency threshold noise reduction is a more effective approach for acoustic signal noise reduction during sic lapping than wavelet packet noise reduction and spectral subtraction noise reduction. This finding has the potential to facilitate the broader application of acoustic emission technology in sic processing. After noise reduction using the frequency threshold noise reduction method, sic lapping acoustic signals can accurately reflect the sic lapping process.

## 2. Materials and Methods

### 2.1. Acoustic Monitoring Platform

**Figure 1:**
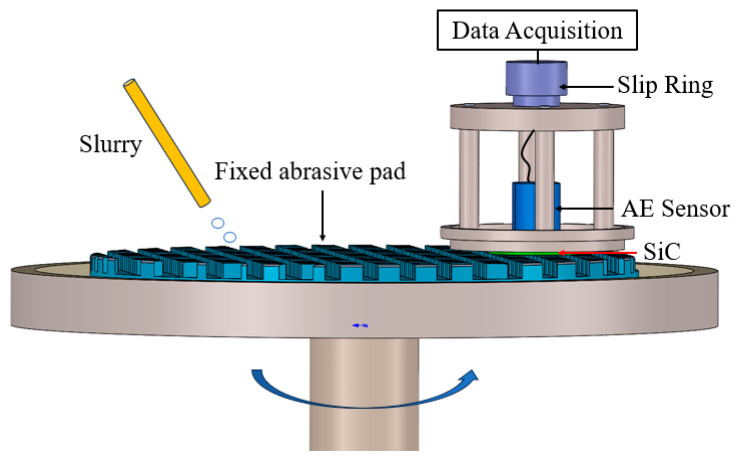
Schematics of the device for AE monitoring of SiC wafer lapping on fixed abrasive pads.

An AE sensor (SAEU3H, QCAE, Guangzhou, China) was mounted on an ultra-precision ring polishing machine (SK-380, Sainko, Dongguan, China) using a self-developed tool to acquire acoustic signals from SiC wafer lapping using a fixed abrasive pad. The sampling rate was set to 6 MHz, and the preamplifier was amplified by 40 dB. The experimental monitoring setup for the lapping process is shown in Figure 1.

### 2.2. Lapping Experiment

**Figure 2:**
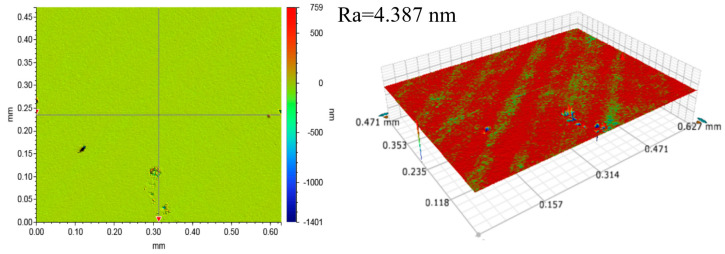
Initial surface morphology of the Si-face of 4H-SiC.

A 4-inch Si-face of 4H-SiC (Jiangyin Lanke Crystal Materials Co., Ltd., Jiangyin, China) was used as a lapping workpiece, and was pre-treated before each set of lapping experiments to achieve a surface roughness of 4 ± 0.5 nm, as shown in Figure 2. The solution used for lapping was deionized water. The lapping pad used in this study is a W40 single crystal diamond fixed abrasive pad provided by Nanjing University of Aeronautics and Astronautics and was trimmed for 5 min before each group of lapping experiments.

The diamond trimmer was used to trim the pad, and the pad was in direct contact with the trimmer during trimming, at speeds of 60 rpm and 45 rpm. The lapping experiment was divided into four sections, and each section of lapping lasted 10 min. Three sets of experiments were performed, and their acoustic signals were collected while the noise acoustic signal was immediately collected after each section of the experiment for 30 s in the stationary state. The specific experiment parameters are shown in Table 1.

The mass of the wafers before and after lapping was measured using an electronic balance with an accuracy of 0.01 mg (OHAUS PMK224 ZH/E, OHAUS, Parsippany, NJ, USA), and the MRR of SiC was then calculated using Equation (1):

(1) $$\mathrm{MRR}=\Delta m/(\rho *\pi *R^{2}*t)$$

where ∆*m* represents the mass difference between the SiC wafer before and after lapping, g; *ρ* is the density of the SiC, g/cm^3^; *R* is the radius of the SiC wafer, cm; and *t* is the lapping time, min.

The RMS of the eigenvalues of the acoustic signal in the time domain was calculated using Equation (2):

(2) $$\mathrm{RMS}=\sqrt{(1/N)*\sum_{i=1}^{N}X_{i}^{2}}$$

where *N* represents the number of sampling points and *x_i_* represents the amplitude corresponding to the *i*-th sampling point.

The acoustic signals were segmented with *L* = 6,000,000 points and analyzed using Python (3.9.1) language code for processing.

The morphology of the lapping pads was examined using a scanning electron microscope (SU8000, Hitachi, Tokyo, Japan). The surface roughness of the SiC was measured with a white light interferometer (Contour GT-K0, Bruker, Billerica, MA, USA).

### 2.3. Data Processing Methods

#### 2.3.1. Wavelet Packet Noise Reduction

Wavelet packet noise reduction can effectively separate the noise when the effective signal frequency band and the noise signal frequency band do not overlap. However, when the frequency bands overlap, noise has to be removed using mathematical models. The mathematical model has two types of noise reduction functions: hard threshold (Equation (3)) and soft threshold (Equation (4)).

Hard threshold function expression:

(3) $$f\left(x\right)=\left\{\begin{array}{c} x,|x|\geq T\\ 0,|x|<T \end{array}\right.$$

Soft threshold function expression:

(4) $$f\left(x\right)=\left\{\begin{array}{ll} sgn(x)(\left|x\right|-T), & |x|\geq T\\ 0, & |x|<T \end{array}\right.$$

where *x* represents the wavelet coefficient and *T* represents the threshold value.

**Figure 3:**
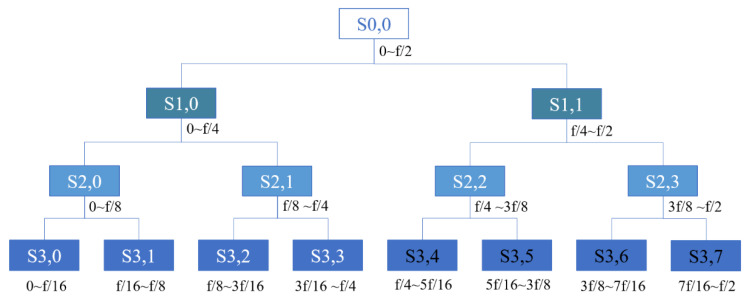
Wavelet packet 3-layer decomposition (*f* is the sampling frequency).

Wavelet packet noise reduction starts with decomposing the original acoustic signal into wavelet coefficients for different frequency bands (Figure 3). Then, the wavelet coefficients of the noise signal frequency band that does not overlap with the effective signal frequency band are identified and removed. Finally, the retained wavelet coefficients are processed using the selected mathematical model. Typically, the wavelet coefficients of the effective signals are significant, while those of the noisy signals are small. Therefore, a threshold value is necessary to distinguish the noise. The *T* (the threshold value) is calculated as the mean of the absolute values of the wavelet packet coefficients of the noise signal (Equation (5)).

(5) $$T=(1/m)*\sum_{n=1}^{m}\left|x_{n}\right|$$

where *m* represents the number of wavelet coefficients and *x_n_* represents the wavelet coefficient of the *n*-th point.

#### 2.3.2. Spectral Subtraction Noise Reduction

Spectral subtraction is a noise reduction technique that is effective for handling smooth noise. In the spectral subtraction noise reduction process, the noise signal and the original signal are first transformed using the fast Fourier transform (FFT) to obtain their respective amplitude spectra (the amplitude distribution of the signal at different frequencies) and phase spectra (the distribution of the phase of the signal at different frequencies), and then, the amplitude spectra of the original signal and the noise signal are then computed using Equation (6) to obtain the amplitude spectra of the effective signal. Finally, the frequency domain effective signal is obtained by combining the amplitude spectrum of the effective signal and the phase spectrum of the original signal, which is then converted to the time domain effective signal using inverse fast Fourier transform (IFFT).

Spectral subtraction noise reduction formula:

(6) $$\left|X\left(w\right)\right|=\left\{\begin{array}{ll} \left|Y\left(w\right)\right|-\left|N\left(w\right)\right|, & \left|Y\left(w\right)\right|-\left|N\left(w\right)\right|\geq 0\\ 0, & \left|Y\left(w\right)\right|-\left|N\left(w\right)\right|<0 \end{array}\right.$$

where *Y*(*w*) represents the original signal amplitude spectrum (with noise); *X*(*w*) represents the effective signal amplitude spectrum (without noise); and *N*(*w*) represents the noise signal amplitude spectrum.

#### 2.3.3. Frequency Threshold Noise Reduction

A frequency threshold noise reduction approach was developed in the study described this manuscript that combines the advantages of wavelet packet noise reduction and spectral subtraction noise reduction. This approach is effective in removing noise regardless of its frequency range and smoothness.

**Figure 4:**
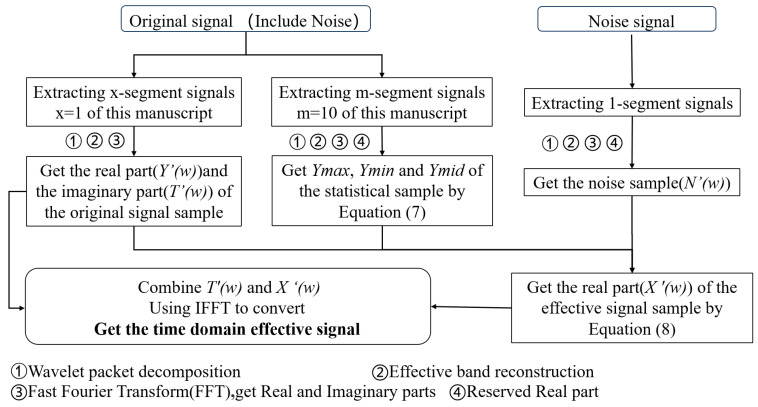
Flowchart of frequency threshold noise reduction.

In this approach, *m* + *x* segments of acoustic signal samples are extracted from the original signals, and one segment of the acoustic signal sample is extracted from the noise signals. These samples are reconstructed after using wavelet packet decomposition to remove unrelated frequency band signals and are then converted to the frequency domain by FFT to obtain the real part (the fundamental amplitude of the signal) and the imaginary part (the relationship between the phase and time of the signal). The statistical acoustic sample is obtained by extracting the real parts of the *m*-segment signal samples and then calculating them using Equation (7). The noise sample(*N*′(*w*)) is the real part of the noise signal sample. The original signal samples are the real parts and the imaginary parts of the *m*-segment signal samples. The original signal sample’s real part is denoted as *Y*′(*w*) and its imaginary part is denoted as *T*′(*w*). The real part of each original signal sample, the statistical sample, and the noise sample are calculated using Equation (8) to obtain the real part(*X*′(*w*)) of the effective signal sample. The frequency domain effective signal is obtained by combining *T*′(*w*) and *X*′(*w*), which are then converted to the time domain effective signal using IFFT. The frequency threshold noise reduction process is presented in Figure 4.

(7) $$\left\{\begin{array}{c} Ymax=\{max\{ai1\},max\{ai2\},\ldots \ldots ,max\{aiw\}\}\\ Ymin=\{min\{ai1\},min\{ai2\},\ldots \ldots ,min\{aiw\}\}\\ Ymid=Ymax-(Ymax-Ymin)/2 \end{array}\right.$$

where *Yi*′(*w*) represents the real parts of the *m*-segment signal samples, *Yi*′(*w*) = {*ai*1, *ai*2, *ai*3, ……, *ai*w}, *i* = 1, 2, 3 …… *m*, and *Ymax*, *Ymin*, and *Ymid* represent the maximum, minimum, and intermediate value samples of the statistical sample, respectively. Let *Ymax*, *Ymin*, and *Ymid* be functions *g*0(*w*), *g*1(*w*), and *g*2(*w*), respectively. Mark *b* = *N*′(*w*)*Y′(*w*), *c* = *Y*′(*w*) − *N*′(*w*), *d* = *Y*′(*w*) − *g*0(*w*), *e* = *Y*′(*w*) − *g*1(*w*).

Frequency threshold noise reduction equation:

(8) $$X^{\prime }\left(w\right)=\left\{\begin{array}{cc} Y^{\prime }\left(w\right)-N^{\prime }(w), & b<0\cup (b\geq 0\cap c\geq 0\cap d\geq 0)\\ Y^{\prime }\left(w\right)+N^{\prime }(w), & b\geq 0\cap (c<0\cup \left(c\geq 0\cap e\leq 0\right))\\ g2(w), & b\geq 0\cap c\geq 0\cap d<0\cap e>0 \end{array}\right.$$

where *X*′(*w*) represents the real part of the effective signal; *N*′(*w*) represents the noise sample; and *Y*′(*w*) represents the original signal sample’s real part.

## 3. Results and Discussion

### 3.1. MRR and Ra of SiC Wafers Lapped on Fixed Abrasive Pads

**Figure 5:**
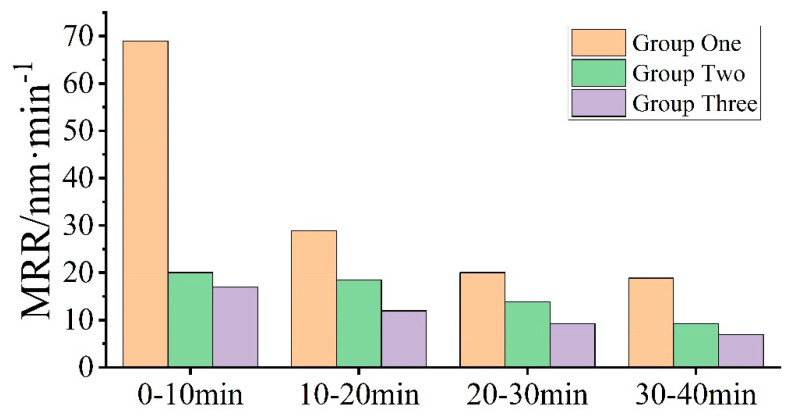
MRR for each segment in the three groups.

**Figure 6:**
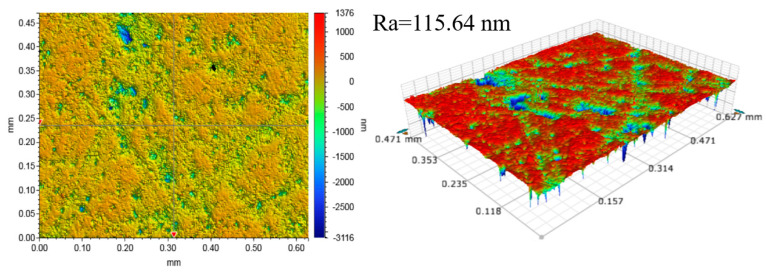
Surface morphology of SiC wafers after the Group One experiment.

**Figure 7:**
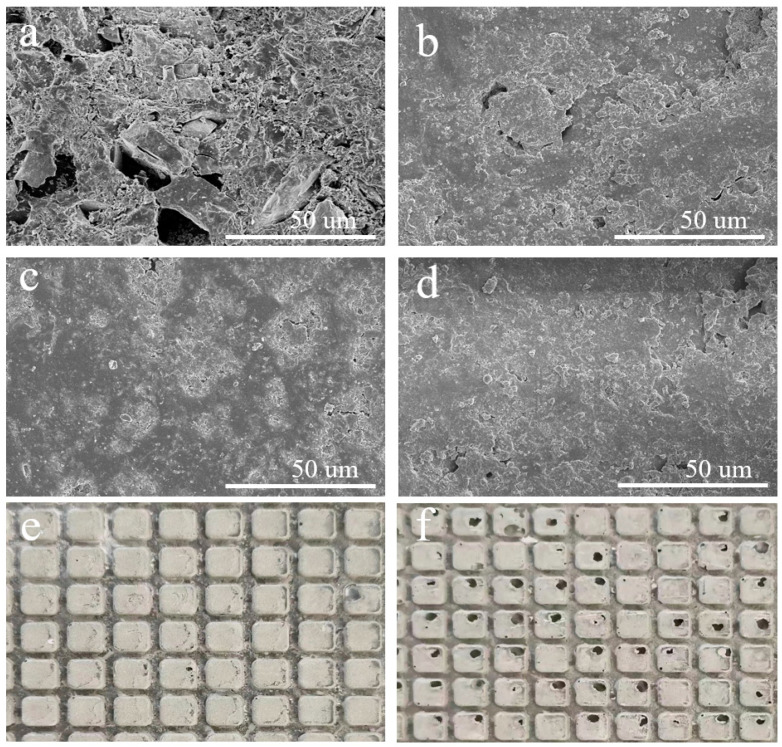
Surface micromorphology of fixed abrasive pads: (**a**) before the Group One experiment; (**b**) after the Group One experiment; (**c**) after the Group Two experiment; (**d**) after the Group Three experiment; macro-morphology of Group One (**e**) and Group Three (**f**).

Figure 5 shows that the MRR decreases with lapping time for all three sets of lapping experiments. By comparing the results in Figure 2 and Figure 6, one can clearly find that the surface scratches on the SiC surface deepen after lapping, which is due to the plastic and elastic deformation of the surface during the lapping process [26], the Ra value increases so that the surface quality deteriorates. The electron microscope SEM image in Figure 7 reveals the surface morphology change of the fixed abrasive lapping pad during the lapping process. Before lapping, abrasive particles on the surface of the fixed abrasive pad are clearly visible, but they cannot be visibly identified on the pad surface as the lapping process continues. This suggests that the particles are either buried underneath or detached from the pad matrix during the lapping process without exposing new abrasive particles, resulting in the MRR decreasing. The MRR of Group One is higher than those of the other two sets, proving that the exposed abrasive particles are the driving force for SiC removal for fixed abrasive pads.

### 3.2. AE Signal Analysis in Lapping

During the lapping process, the acoustic signal comprises an effective signal and a noise signal. Hase [27] and Chen [28] categorized the frequency bands of AE signals based on material removal mechanisms. In lapping processes, the main processing acoustic frequency bands fall into a range within 1 MHz. Therefore, this manuscript will focus on analyzing the frequency band between 0~1.125 MHz. The remaining frequency band signals are considered process noise.

The noise signal originates from several sources, including mechanical vibration noise generated by the rotating polishing machine, shaking noise from tooling, noise from line interference during signal transmission, and surrounding environmental noise. However, the rotational characteristics of the polishing machine prevent the lapping pad and the workpiece from achieving a completely static state during rotation. Therefore, only the noise generated during the polishing machine’s static state can be collected. This noise includes noise from line interference during signal transmission and environmental noise.

**Figure 8:**
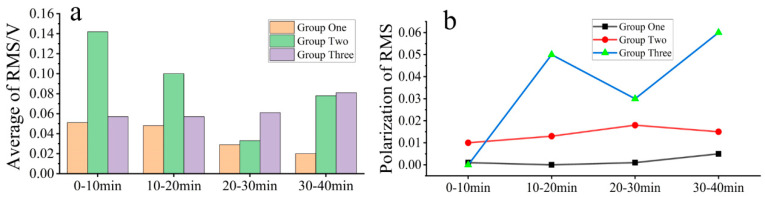
RMS of samples per noise segment; (**a**) average of RMS and (**b**) polarization of RMS.

For each segment of the lapping process, three segments of the ‘L’ signal were selected for noise signal analysis. Before it can be used for frequency threshold noise reduction, the original noise signal is reconstructed after using wavelet packet decomposition to remove unrelated frequency noise in the lapping process to obtain the valid frequency band signal, and the RMS values of the valid frequency band signals were calculated and are presented in Table 2, Table 3 and Table 4. Based on the RMS values in these tables, the mean and extreme deviation of the noise signal samples for each segment were calculated as shown in Figure 8. Figure 8a shows that the mean values of RMS values are all different and vary irregularly, indicating that there is randomness in the noise fluctuation, while the mean value in Group Two is larger than that in the other two groups, indicating that the noise fluctuation in Group Two is the largest among the three groups of experiments. As depicted in Figure 8b, Group Two exhibits a significantly higher extreme difference value compared to the other two groups, suggesting greater variation in noise in each section experiment of this group. These results all suggest that the noise appears randomly without any regularity and fluctuates the most in Group Two of the experiments.

For each segment of the lapping process, one segment of the ‘L’ signal was selected for lapping signal analysis, and its RMS values (original AE signal) were calculated. To better extract acoustic signals of the lapping process, four noise reduction approaches, which are wavelet packet hard threshold, wavelet packet soft threshold, spectral subtraction, and frequency threshold, were applied to the original acoustic signals, and their RMS values of lapping signal after using noise reduction approaches were calculated. These RMS values are presented in Table 5, Table 6 and Table 7.

### 3.3. Comparison of Noise Reduction Methods

**Figure 9:**
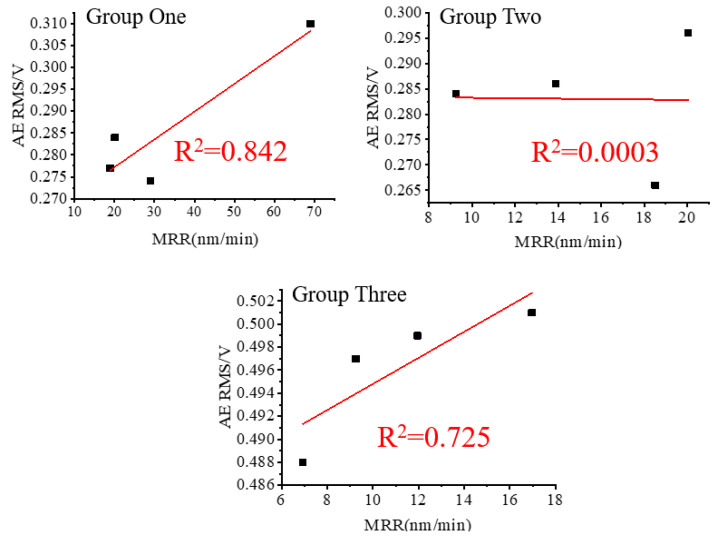
Original AE RMS and MRR.

The RMS values (original AE signal) were plotted against the values of the MRR and then linearly fitted to the values of the MRR of the three sets of lapping experiments in Figure 9. As evidenced in Figure 9, the RMS values of Groups One and Three show a better linear fit with the values of the MRR than that of Group Two. Taking into consideration the noise influence shown in Figure 8, one can find that the noise RMS values of Groups One and Three are smaller and smoother, which might be the reason for resulting in a larger R^2^. Conversely, the noise MRS value of Group Two is larger, resulting in an R^2^ close to 0. This suggests that noise fluctuation has a significant impact on the accurate measurement of lapping acoustic signals.

**Figure 10:**
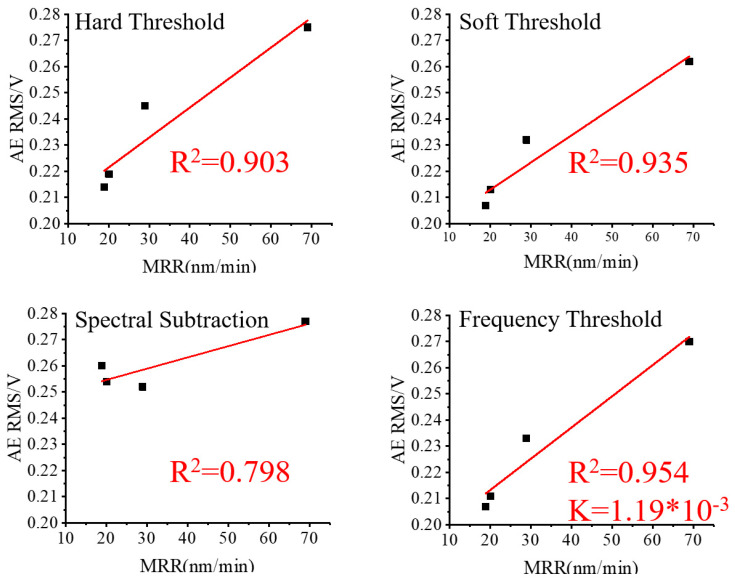
Group One AE RMS after noise reduction and MRR.

**Figure 11:**
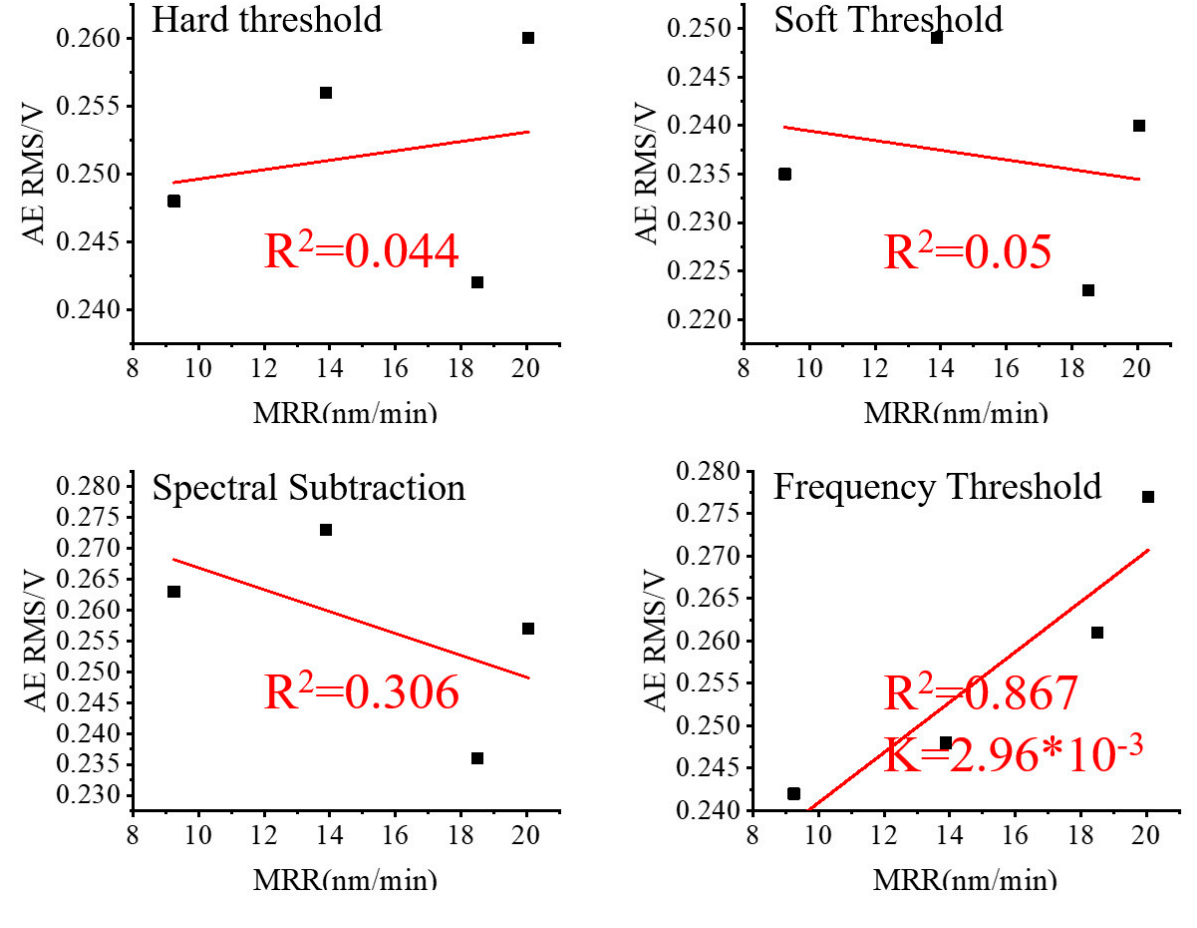
Group Two AE RMS after noise reduction and MRR.

**Figure 12:**
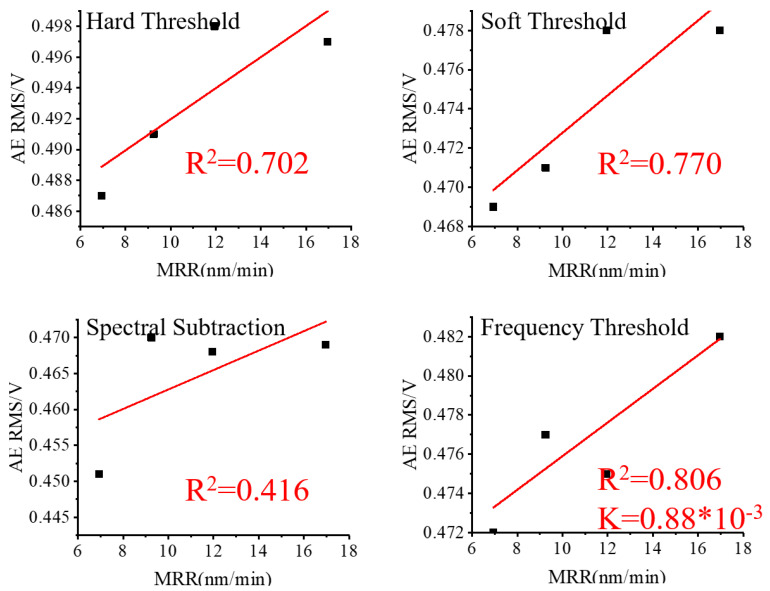
Group Three AE RMS after noise reduction and MRR.

To analyze the impact of various noise reduction methods, the RMS values of the three sets of lapping signals after noise reduction were linearly fitted to their corresponding values of MRR and plotted in Figure 10, Figure 11 and Figure 12.

Based on Figure 9 and Figure 10, it is evident that the R^2^ improved in Group One after applying the wavelet packet noise reduction and frequency threshold noise reduction methods, and worsened, however, after applying the spectral subtraction noise reduction method. In comparison, the frequency threshold noise reduction method produced the highest R^2^ and the most favorable results among the three noise reduction methods. Based on Figure 11, the R^2^ obtained through the wavelet packet noise reduction and spectral subtraction noise reduction methods in Group Two are the worst. Only after applying the frequency threshold noise reduction method is a reasonably improved R^2^ achieved. Therefore, the frequency threshold noise reduction method is the most effective for the Group Two data set. Based on Figure 9 and Figure 12, it is evident that R^2^ in Group Three was improved by using wavelet packet soft threshold noise reduction and frequency threshold noise reduction. The other methods deteriorate R^2^.

In summary, for all three sets of data, there is no doubt that the frequency threshold noise reduction method is most effective in reducing the impact of acoustic signal noise in the lapping process for both stable and fluctuating noise situations. For stable noise fluctuations, several noise reduction methods may be used, but their effectiveness is limited and sometimes counterproductive. For large noise fluctuations, only frequency threshold noise reduction is effective, and other noise reduction methods are not applicable.

Figure 10, Figure 11 and Figure 12 show the linear fitting of MRR and RMS in each group after using frequency threshold noise reduction. The slopes (K) in the plots are all different, indicating that the linear fitting equations are different for each group. A comparative analysis was conducted between Groups One and Three. There are three main removal mechanisms in the lapping process: rubbing, plowing, and cutting. The RMS and frequency bands of the acoustic signals differ for each mode. According to Chen’s [28] frequency domain division approach, the frequency band is categorized as 0~0.2 MHz for sliding, 0.05~0.7 MHz for plowing, and 0.5~1.125 MHz for cutting. The frequency domain feature information is calculated using the following formula:

(9) $$\left\{\begin{array}{c} Px=\sum_{k=0}^{2*10^{5}}\left|A_{k}\right|\\ Py=\sum_{k=5*10^{4}}^{7*10^{5}}\left|A_{k}\right|\\ Pz=\sum_{k=5*10^{5}}^{1.125*10^{6}}\left|A_{k}\right| \end{array}\right.$$

where *A_k_* represents the amplitude corresponding to the *k* frequency in the frequency domain of the sample and *Px*, *Py*, and *Pz* represent the sum of the absolute values of the amplitudes in different frequency bands.

**Figure 13:**
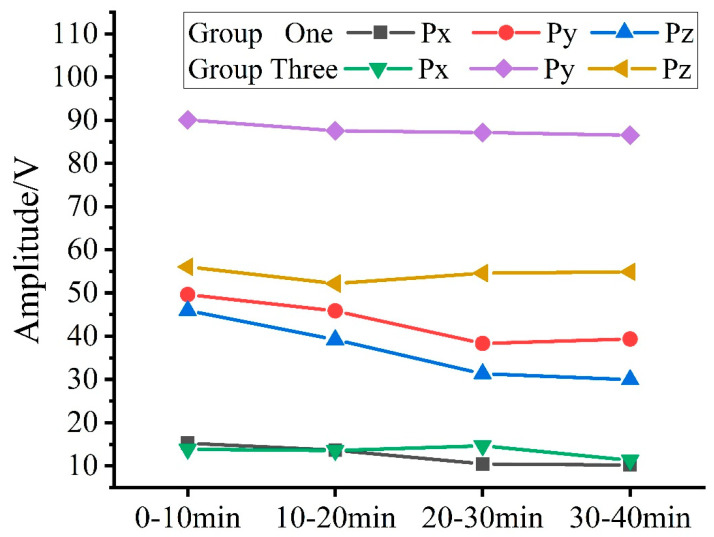
Groups One and Three of frequency domain features.

Figure 13 shows the frequency domain feature information of the AE signals of Groups One and Three after using frequency threshold noise reduction. The *Px* values are similar, while the *Py* and *Pz* values are significantly higher in Group Three, indicating more plowing and cutting during SiC lapping. However, as shown in Figure 5, the MRR of Group Three is lower than that of Group One. Therefore, the increase in *Py* and *Pz* values does not contribute to an increase in MRR. Figure 7e,f illustrates that the surface of the pad during Group One of lapping is flat while during Group Three of lapping, a large number of pits are found on the surface of the pad, revealing a surface morphology change of the polishing pads from good to bad. The primary factor contributing to this deterioration in surface morphology is fixed abrasive pad trimming.

The diamond trimmer is in direct and immediate contact with the fixed abrasive pad, sharpening the surface abrasive grains of the fixed abrasive pad. Additionally, the process results in the shedding of some of the abrasive grains of the fixed abrasive pad. The RMS values of Group Three are significantly higher than those of Group One, as shown in Figure 10 and Figure 12, while the values of the MRR are smaller. This inconsistency is mainly due to the deterioration in the state of the abrasive pads, which leads to more noise generation but not necessarily higher MRR. As a result, the linear fitting curves for each group should be carried out independently. If the linear equation between RMS and MRR is not re-established after the fixed polishing pad is trimmed and the previous equation is used, the MRR values predicted using RMS will be significantly off and will not accurately reflect the lapping process.

## 4. Conclusions

During the fixed abrasive lapping of SiC wafers, randomly generated noise can significantly reduce the measurement accuracy of acoustic signal monitoring. In the study described in this manuscript, a frequency threshold noise reduction approach was developed, and its effectiveness in acoustic emission monitoring of the SiC lapping process was compared with wavelet packet noise reduction and spectral reduction methods. The following conclusions for the three noise reduction methods were reached:

(1) For smooth noise, the wavelet packet noise reduction method and the frequency threshold noise reduction method can effectively reduce the noise, while the spectral method noise reduction method cannot effectively reduce the noise. The frequency threshold noise reduction method performs the best in smooth noise situations.
(2) For fluctuating noise, only the frequency threshold noise reduction method can effectively reduce the noise while the wavelet packet noise reduction method and the spectral reduction method are ineffective.
(3) The frequency threshold noise reduction method is effective for both smooth and fluctuating noise.
(4) The lapping pad trimming process degrades the macroscopic morphology of the lapping pad surface, resulting in noise degradation. It is necessary to re-establish the linear fitting laws for RMS and MRR after each lapping pad trimming.

## Figures and Tables

**Table 1:** Lapping process parameters.

Process Parameters	Conditions
Workpiece	Si-face of 4H–SiC, 4-inch
Pad	Fixed abrasive pad
Pressure	9.7 kPa
Slurry flow rate	20 mL/min
Pad/wafer rotational speed	60/45 r/min
Lapping time	10 min

**Table 2:** RMS of Group One noise.

Group One	0–10 min	10–20 min	20–30 min	30–40 min
RMS (Sample 1)	0.043	0.045	0.048	0.020
RMS (Sample 2)	0.067	0.056	0.020	0.020
RMS (Sample 3)	0.042	0.043	0.020	0.020

**Table 3:** RMS of Group Two noise.

Group Two	0–10 min	10–20 min	20–30 min	30–40 min
RMS (Sample 1)	0.142	0.139	0.023	0.147
RMS (Sample 2)	0.142	0.136	0.023	0.044
RMS (Sample 3)	0.142	0.024	0.052	0.044

**Table 4:** RMS of Group Three noise.

Group Three	0–10 min	10–20 min	20–30 min	30–40 min
RMS (Sample 1)	0.056	0.057	0..061	0.112
RMS (Sample 2)	0.057	0.057	0.062	0.064
RMS (Sample 3)	0.057	0.057	0.061	0.066

**Table 5:** RMS values of Group One original and noise-cancelled signals.

Group One	0–10 min	10–20 min	20–30 min	30–40 min
RMS (Original Signal)	0.310	0.274	0.284	0.277
RMS (Hard Threshold)	0.275	0.245	0.219	0.214
RMS (Soft Threshold)	0.262	0.232	0.213	0.207
RMS (Spectral Subtraction)	0.277	0.252	0.254	0.260
RMS (Frequency Threshold)	0.270	0.233	0.211	0.207

**Table 6:** RMS values of Group Two original and noise-cancelled signals.

Group Two	0–10 min	10–20 min	20–30 min	30–40 min
RMS (Original Signal)	0.296	0.266	0.286	0.284
RMS (Hard Threshold)	0.260	0.242	0.256	0.248
RMS (Soft Threshold)	0.240	0.223	0.249	0.235
RMS (Spectral Subtraction)	0.257	0.236	0.273	0.276
RMS (Frequency Threshold)	0.277	0.261	0.248	0.242

**Table 7:** RMS values of Group Three original and noise-cancelled signals.

Group Three	0–10 min	10–20 min	20–30 min	30–40 min
RMS (Original Signal)	0.501	0.499	0.497	0.488
RMS (Hard Threshold)	0.497	0.498	0.491	0.487
RMS (Soft Threshold)	0.478	0.478	0.471	0.469
RMS (Spectral Subtraction)	0.469	0.468	0.470	0.451
RMS (Frequency Threshold)	0.482	0.475	0.477	0.472

## Data Availability

The original contributions presented in the study are included in the article, further inquiries can be directed to the corresponding authors.

## References

[1] Sun K., Wang T., Gong W.B., Lu W.Y., He X., Eddings E.G., Fan M. (2022). Synthesis and potential applications of silicon carbide nanomaterials/nanocomposites. Ceram. Int..

[2] She X., Huang A., Lucia O., Ozpineci B. (2017). Review of Silicon Carbide Power Devices and Their Appons. IEEE Trans. Ind. Electron..

[3] Wang W., Lu X., Wu X., Zhang Y., Wang R., Yang D., Pi X. (2023). Chemical–Mechanical Polishing of 4H Silicon Carbide Wafers. Adv. Mater. Interfaces.

[4] Ou Y., Lan Z., Hu X., Liu D. (2024). Novel SiC Trench MOSFET with Improved Third-Quadrant Performance and Switching Speed. Micromachines.

[5] Chen J., Peng N. (2023). Super hard and brittle material removal mechanism in fixed abrasive lapping: Theory and modeling. Tribol. Int..

[6] Lu J., Li Y., Xu X. (2015). The effects of abrasive yielding on the polishing of SiC wafers using a semi-fixed flexible pad. Proc. Inst. Mech. Eng. Part B J. Manuf. Syst..

[7] Zhou P., Li J., Wang Z., Chen J., Li X., Zhu Y. (2020). Molecular dynamics study of the removal mechanism of SiC in a fixed abrasive polishing in water lubrication. Ceram. Int..

[8] Xie Y., Bhushan B. (1996). Effects of particle size, polishing pad and contact pressure in free abrasive polishing. Wear.

[9] Chen J., Peng Y., Wang Z., Sun T., Su J., Zuo D., Zhu Y. (2022). Tribological effects of loose alumina abrasive assisted sapphire lapping by a fixed agglomerated diamond abrasive pad (FADAP). Mater. Sci. Semicond. Process..

[10] Hsieh C.H., Chang C.Y., Hsiao Y.K., Chen C.C.A., Tu C.C., Kuo H.C. (2022). Recent Advances In Silicon Carbide Chemical Mechanical Polishing Technologies. Micromachines.

[11] Li G., Bao Y., Wang H., Dong Z., Guo X., Kang R. (2023). An online monitoring methodology for grinding state identification based on real-time signal of CNC grinding machine. Mech. Syst. Signal Process..

[12] Xu C., Guo D.M., Kang R.K., Jin Z.J., Huo F.W. (2008). Friction-based in situ endpoint detection of copper CMP process. Adv. Mater. Res..

[13] Hocheng H., Huang Y. (2004). In Situ Endpoint Detection by Pad Temperature in Chemical–Mechanical Polishing of Copper Overlay. IEEE Trans. Semicond. Manuf..

[14] He A., Liu B., Song Z., Liu W., Lu Y., Wang L., Wu G., Feng S. (2013). Endpoint detection of Ge2Sb2Te5 during chemical mechanical planarization. Appl. Surf. Sci..

[15] Fujita T., Kitade K. (2019). Development of endpoint detection using optical transmittance and magnetic permeability based on skin effect in chemical mechanical planarization. Precis. Eng..

[16] Helu M., Chien J., Dornfeld D. (2014). In-situ CMP Endpoint Detection Using Acoustic Emission. Procedia CIRP.

[17] Ahn B., Lee S. (2009). Characterization and acoustic emission monitoring of AFM nanomachining. J. Micromech. Microeng..

[18] Liu C., Chen H., Lin S. (2019). Acoustic Emission Monitoring System for Hard Polishing of Sapphire Wafer. Sens. Mater..

[19] Pardo E., San Emeterio J.L., Rodriguez M.A., Ramos A. (2006). Noise reduction in ultrasonic NDT using undecimated wavelet transforms. Ultrasonics.

[20] Lmgr M., Guo H., Odegard J.E., Burrus C.S., Wells R.O. (1996). Noise reduction using an undecimated discrete wavelet transform. IEEE Signal Process. Lett..

[21] Yang Y., Li S., Li C., He H., Zhang Q. (2022). Research on ultrasonic signal processing algorithm based on CEEMDAN joint wavelet packet thresholding. Measurement.

[22] Bettayeba F., Hacianeb S., Aoudiab S. (2005). Improving the time resolution and signal noise ratio of ultrasonic testing of welds by the wavelet packet. NDT E Int..

[23] Boll S. (1979). Suppression of Acoustic Noise in Speech Using Spectral Subtraction. IEEE Trans. Acoust. Speech Signal Process..

[24] Paliwal K., Wójcicki K., Schwerin B. (2010). Single-channel speech enhancement using spectral subtraction in the short-time modulation domain. Speech Commun..

[25] Sikder A., Gitis N., Vinogradov M., Daugela A. (2004). In-situ tribological properties monitoring and chemical mechanical characterization of planarization process. Int. Jt. Tribol. Conf..

[26] Dang J., Wang H., Wang C., An Q., Li Y., Wang H., Chen M. (2024). Microstructure evolution and surface strengthening behavior of 300 M ultrahigh strength steel under engineered surface treatments. Mater. Charact..

[27] Hase A., Mishina H., Wada M. (2012). Correlation between features of acoustic emission signals and mechanical wear mechanisms. Wear.

[28] Chen J., Li J., Peng Y., Wang Z., Sun T., Zhu Y. (2022). In-situ manifestation of lapping mechanisms by rapid intelligent pattern recognition analysis (RIPRA) of acoustic emission via a point density fuzzy C-means (PD-FCM) method. J. Manuf. Process..

